# The Population Ecological Characteristics of Gongshan Muntjac (*Muntiacus gongshanensis*) in Southeastern Tibet Based on Camera‐Trap Technology

**DOI:** 10.1002/ece3.71646

**Published:** 2025-07-01

**Authors:** Qianqian Wang, Biao Yang, Jin Chang, Xin Wang, Xiaoguo Chen, Shilin Li, Jiangcuo Renzeng, Dunzhu Gongqiu, Li Zhang

**Affiliations:** ^1^ Key Laboratory of Biodiversity and Ecological Engineering, Ministry of Education, College of Life Sciences Beijing Normal University Beijing China; ^2^ College of Life Sciences China West Normal University Nanchong China; ^3^ Society of Entrepreneurs and Ecology (SEE) Foundation Beijing China; ^4^ Sichuan Zoological Society Chengdu China; ^5^ Southwest Forestry University Kunming China; ^6^ Gedang Public Welfare Forest Professional Management and Protection Station Linzhi China; ^7^ China Civil Affairs University Beijing China

**Keywords:** activity patterns, camera trap survey, habitat use, *Muntiacus gongshanensis*, sex ratio, Tibetan biodiversity

## Abstract

The Gongshan muntjac ( *Muntiacus gongshanensis* ) is one of the least understood ungulate species and is classified as Critically Endangered (CR) on China's Vertebrates Red List and assessed as Data Deficient (DD) by the IUCN. Its elusive behavior, remote habitat, and restricted distribution have hindered efforts to understand its ecological characteristics. To address this gap, we conducted an extensive camera trap survey in Gedang, Medog County, Southeast Tibet, from April 2023 to December 2024. A total of 4846 images and videos from 914 independent detections were collected from 52 cameras that captured the Gongshan muntjac. The results revealed that Gongshan muntjac was widely distributed at elevations below 2800 m, with particularly frequent habitat use below 2200 m in broadleaf forests. The observed sex ratio favored males, with a female‐to‐male sex ratio of 1:1.13 in this wild population. Solitary individuals were the most common social structure (89.71% of independent detections), followed by female–male pairs (6.94%), whereas other groups with limited detections altogether accounted for only 3.35%. The daily activity patterns followed a crepuscular bimodal rhythm, with the primary activity intensity concentrated at dusk (19:00–21:00) and a smaller peak at dawn (07:00–09:00), and there were no significant differences between males and females. Although activity frequencies were higher in summer and autumn, daily activity intensity exhibited no significant seasonal variation. These findings contribute critical baseline data on the habitat preferences, activity rhythms, and population structure of Gongshan muntjac, offering valuable guidance for conservation planning and future ecological monitoring of this poorly understood species.

## Graphical abstract

This study provides critical insights into the ecological characteristics of the endangered Gongshan muntjac, a poorly understood ungulate species. Our findings reveal the species' habitat preferences, activity rhythms, and social structure, which are vital for informing conservation strategies and enhancing our understanding of this species' role in its ecosystem. The research contributes to the scientific basis for protecting the Gongshan muntjac and offers a model for studying other elusive wildlife.

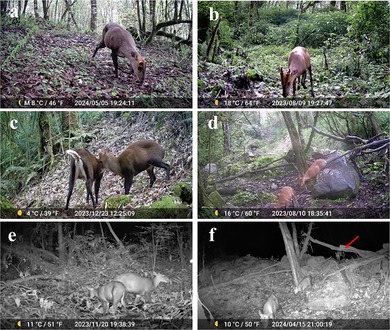

## 1 Introduction

The dynamics and evolution of animal populations are significantly influenced by factors, such as social structure, sex ratio, and activity patterns (Bond et al. 2019). Among ungulates, the diversity of social structures is crucial for individual survival rates, foraging efficiency, and mate selection (Scott et al. 2014; Liu et al. 2021). These varied social assemblages enhance adaptation to the environment and provide strategic responses to predatory threats and fluctuations in resource availability (Sah et al. 2017; Liu et al. 2021). Additionally, investigating the specific activity patterns of species is essential for understanding their environmental adaptation strategies, preferred habitats, and the scope of their ecological niches (Lu et al. 2007; Ridout and Linkie 2009; Azevedo et al. 2018).

The genus *Muntiacus*, a medium‐ to small‐sized herbivorous animal belonging to the family Cervidae under the subfamily Muntiacinae in the order Artiodactyla, exhibits high species diversity, with 13 species currently described (Yin et al. 2021; Liu et al. 2022; He et al. 2024). In China, several species of the genus *Muntiacus* have been recorded, including Gongshan muntjac ( *Muntiacus gongshanensis* ), Reeves' muntjac ( *M. reevesi* ), Northern red muntjac ( *M. vaginalis* ), Black muntjac ( *M. crinifrons* ), Fea's muntjac ( *M. feae* ), and Leaf muntjac ( *M. putaoensis* ) (Ma et al. 1986; Huang et al. 2006; Timmins and Duckworth 2016; Chen et al. 2022). Among these species, the Gongshan muntjac is particularly notable because of its relatively recent discovery and classification. This medium‐sized Muntiacus species was initially studied and identified through chromosome differences by Shi and Ma (1988) and was subsequently characterized by Ma et al. (1990) based on specimens obtained from Yunnan. This species features dark brown coloration on its ventral body parts, extending to the dorsal surface of the tail, with dark black coloration on the lateral sides and limbs, whereas the axillary, inguinal, inner thigh, and perianal area as well as the ventral surface of the tail are white. However, the lack of comprehensive scientific inquiry may render existing characterizations of the Gongshan muntjac's habitat and activity patterns potentially unreliable.

Current studies indicate that the habitat of the Gongshan muntjac in China spans the Northwestern region of Yunnan, including Gongshan, Fugong, and Tengchong, and extends to Bomi in Southeastern Tibet, as well as reaching Northern Myanmar and Northeastern India (Choudhury 2009; Aiyadurai and Meme 2015; Timmins and Duckworth 2016; Huang et al. 2019; He et al. 2024). The conservation status of the species is classified as Critically Endangered (CR) on China's Vertebrates Red List Status by Jiang et al. (2016) and as Data Deficient (DD) on the IUCN (2025) Red List of Threatened Species. As one of the least studied ungulate species, there is a notable lack of research on the Gongshan muntjac, both domestically and internationally, mainly due to its late discovery, restricted distribution range, and elusive nature, all of which complicate research efforts (Zhang et al. 2019; Yin et al. 2021; Liu et al. 2022; Wang et al. 2022). This neglect is particularly concerning given the impacts of rapid habitat changes and potential climate change on its survival, underscoring the urgent need for increased scientific investigations and research efforts to better understand and conserve this species.

Medog, located in Southeastern Tibet, is recognized as one of the 36 biodiversity hotspots (Myers et al. 2000; Shi et al. 2023). It is characterized by extensive forest resources and coverage, substantial elevational variation, and abundant rainfall, all of which are pivotal for maintaining ecosystem integrity, providing habitats for a variety of wildlife, including the Gongshan muntjac (Wen et al. 2014; Ren et al. 2024). However, due to the challenges associated with implementing traditional survey methods in its residential area, the ecological characteristics of the Gongshan muntjac remain poorly documented. With the rise of camera trap technology, unattended and noninjurious wildlife observations have become more effective, allowing for continuous monitoring around the clock in natural environments and playing a crucial role in the study of elusive, nocturnal, and highly vigilant species (Li et al. 2014; Rowcliffe et al. 2014; Pyšková et al. 2018). Data from infrared camera traps provide valuable insights into wildlife ecology, including species distribution (Wevers et al. 2021), population density (Zampetti et al. 2024), and behavior (Earl et al. 2024).

Therefore, this study aims to fill these critical knowledge gaps by employing long‐term surveillance using infrared camera‐trap technology. Through statistical analysis of the imagery data, the objectives are to assess the habitat distribution, sex ratio, and social structure patterns of the population, and analyze the daily activity rhythms and seasonal differences of the Gongshan muntjac. These results provide essential baseline data for the conservation and management of this elusive species, elucidate a comprehensive understanding of its ecological characteristics, and substantially support the development of effective conservation strategies.

## 2 Material and Methods

### 2.1 Study Area

**FIGURE 1:**
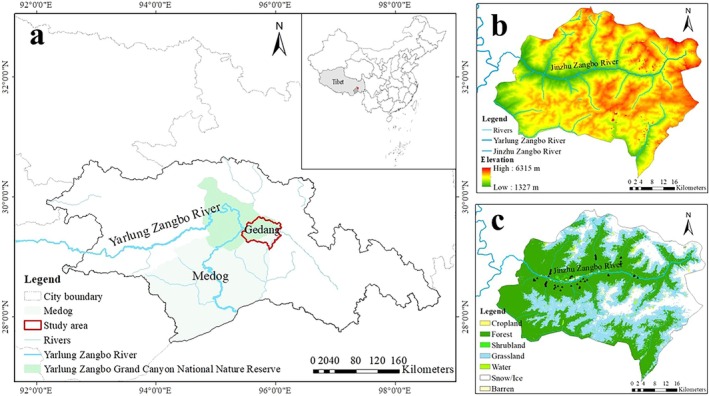
(a) Location map of Gedang, Southeastern Tibet; (b) digital elevation model (DEM) derived from 1:1 million data, provided by the National Catalog Service for Geographic Information (https://www.webmap.cn/main.do?method = index); and (c) land use types, as derived from the 30 m annual land cover dataset from the Geospatial Data Cloud (https://www.gscloud.cn/search), with the black dots representing the sites of camera traps.

Gedang, located within Medog in the Tibet Autonomous Region of China, is a vital component of the Yarlung Zangbo Grand Canyon National Nature Reserve, with the Yarlung Zangbo River flowing from north to south throughout the territory. This township covers more than 2000 km^2^, with an elevation ranging from 1327 to 6315 m (Figure 1). The barrier effect of the Himalayas, along with diverse topography and complex geological conditions, has fostered a unique ecological environment that supports a wide range of vertical vegetation zones, from tropical rainforests to cold alpine vegetation (Wen et al. 2014; Weng et al. 2022). These zones include coniferous forests, broadleaf forests, mixed forests, shrublands, and alpine vegetation, reflecting a gradient from subtropical to tropical mountain regions and fostering an extraordinary level of biodiversity, providing habitats for diverse wildlife (Wu et al. 2016; Shi et al. 2023). Some notable wild ungulate species found in the area include Takin ( *Budorcas taxicolor* ), Red goral ( *Naemorhedus baileyi* ), Himalayan serow ( *Capricornis thar* ), Black musk deer ( *Moschus fuscus* ), and the recently reported Gongshan muntjac in Tibet (Wang et al. 2022; Wang et al. 2024). Among these, the Gongshan muntjac, an herbivore that aids in seed dispersal and serves as prey for larger predators, is particularly noteworthy as the only species of the genus *Muntiacus* in the region, holding significant ecological importance and conservation value.

### 2.2 Infrared Camera Trap Survey

From April 2023 to November 2024, over 150 infrared cameras (MT 660 series, Shenzhen Zhongye Yunhu Electronic Equipment Co. Ltd., Shenzhen, China) were deployed across various habitats within Gedang, covering an elevation range from 1798 m to 3654 m. The cameras were set to capture 3 photographs followed by a 20 s video, with a 30 s interval between triggers, and configured for continuous 24 h operation. To minimize irrelevant triggers, the camera sensitivity was set to “medium”. The deployment strategy included a grid layout in large areas, using geographic information system (ArcGIS) technology to divide areas with concentrated coverage into 1 × 1 km grids, with one or two cameras placed in each grid, and a hybrid approach that combined random and linear deployment methods in elongated areas. Additionally, the distance between two neighboring cameras was no less than 500 m (Deng et al. 2021). Deployment sites were selected based on high animal activity and clear signs of animal presence, such as trails, water sources, and feces. Cameras were securely mounted on suitable tree trunks at approximately 0.4–0.6 m above ground level, with adjustments to the shooting angle to avoid direct sunlight, backlighting, and obstructions. After installation, test shots were taken to verify functionality, and detailed records, including camera numbers, GPS coordinates, and microhabitat characteristics, were recorded for each site (Xiao et al. 2014; Wang et al. 2024). Batteries and SD cards were replaced every 3–5 months, and in cases of camera malfunction, immediate repair or replacement was conducted; for sites where no wildlife was detected or where cameras were lost, new appropriate locations within the suitable habitat were selected, with each camera operating for a minimum of 100 camera days to ensure continuous monitoring and reliable data collection.

### 2.3 Data Analysis

#### 2.3.1 Infrared Camera Data Compilation and Analysis

The valid images and videos captured by the camera traps were organized and managed using Bio‐Photo (V2.1) software, according to the date and time of capture. Invalid images and videos that did not feature animals or humans were discarded, and the identification of mammalian species followed the criteria described by Liu et al. (2022), Smith and Xie (2009), and Jiang et al. (2015, 2017).

A camera day is defined as a continuous 24 h period of operation for an infrared camera at a single location in the wild. For continuous imagery that enabled the identification of individuals or groups of species, each detection of a separate individual or group was treated as an independent detection. For imagery that did not facilitate such identification, independent detection was defined as the first capture of a species within a 30 min interval following the previous capture (O'Brien et al. 2003).

#### 2.3.2 Sex Identification Methods

**FIGURE 2:**
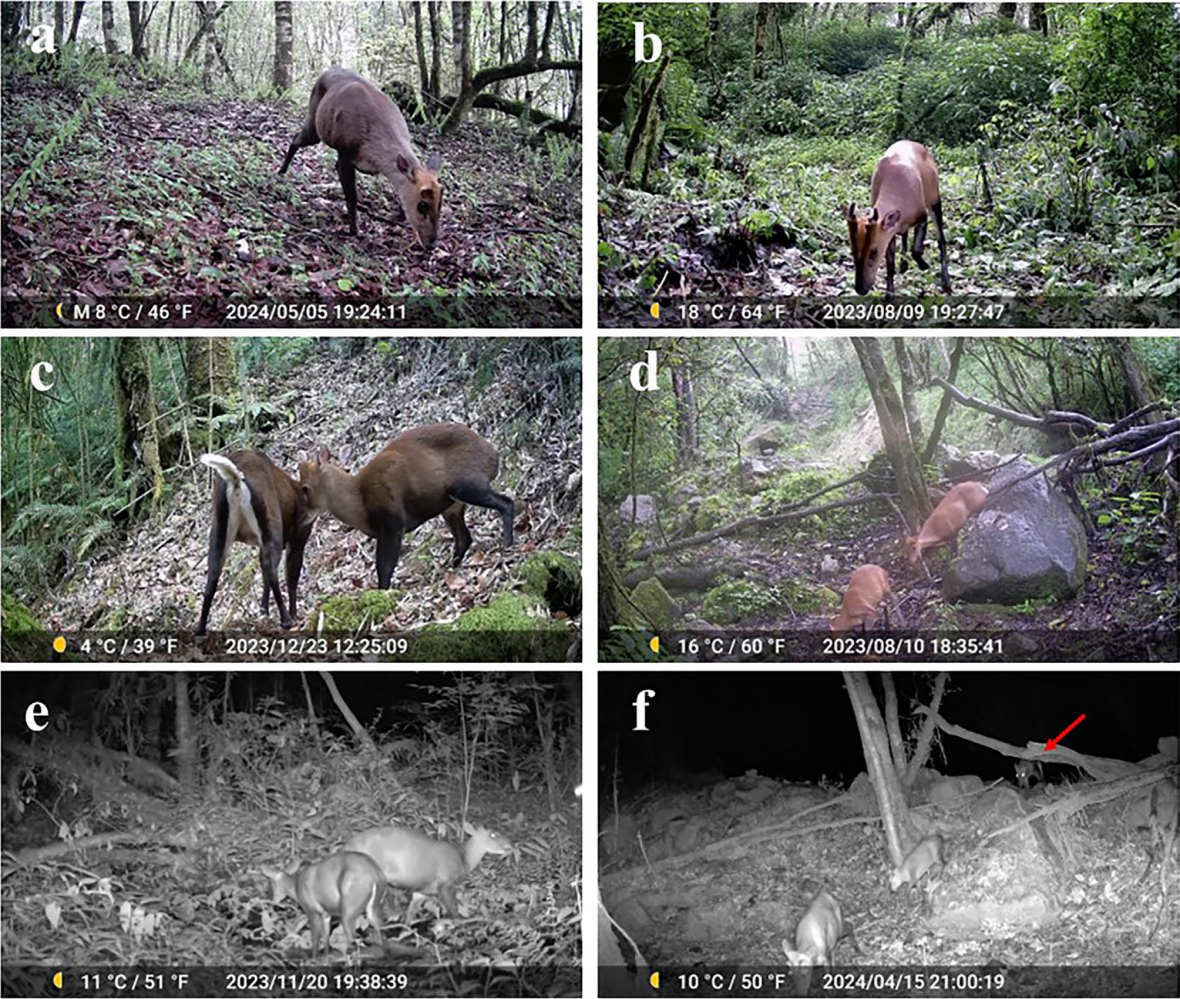
Adult female and male individuals and typical group types of *Muntiacus gongshanensis* . (a) Solitary adult female; (b) Solitary adult male; (c) Female–male pair; (d) Female pair; (e) Doe‐fawn group; and (f) Family group.

The sex determination of adult Gongshan muntjac relies primarily on the presence or absence of antlers (Figure 2). Adult males are distinguished by a pair of antlers, which may be either single or bifurcated. These antlers are short and robust, with particularly short pedicles covered with black fur at the front end. The pedicles extend downward to form a ridge‐like bony protrusion on the skull, creating a V‐shape on the forehead. In contrast, adult females lack antlers, but the V‐shaped black pattern on the forehead is equally distinct (Liu et al. 2022). During the count of adult female and male individuals in a single independent detection, if the sex of one or more individuals could not be determined, the entire detection was excluded. Fawn individuals are distinguished from adults by their smaller body size, but other characteristics are difficult to assess from images and videos because of the lack of relevant records; thus, developmental stages, such as subadults or juveniles were not included in this study.

#### 2.3.3 Social Structure Identification

Consistent with the grouping patterns of muntjac species described by Chen et al. (2022), the criteria for categorizing different group patterns among the Gongshan muntjac were as follows: (1) Solitary adult female: A single adult female was observed independently, without other individuals; (2) Solitary adult male: A single adult male was observed independently, without other individuals; (3) Female group: A group consisting of at least two females was present at the same site; (4) Male group: A group consisting of at least two males was present at the same site; (5) Mixed female–male group: A group consisting of at least one male and one female was observed, which could be a female–male pair or a group with more than two individuals of each sex present at the same site, such as one male‐two female group; (6) Doe/Buck‐fawn group: A group consisting of at least one adult and one fawn was present at the same site; (7) Family group: A group consisting of at least one adult male, one adult female, and one fawn was present at the same site (Figure 2).

#### 2.3.4 Relative Abundance Index

The relative activity indices (RAIs) were quantified as the number of detections per 1000 camera days to analyze the monthly and annual activity patterns of the Gongshan muntjac (Bu et al. 2016). The seasonal relative abundance index (sRAI) and monthly relative abundance index (mRAI) were calculated based on independent detections, providing valuable insights into the population dynamics of the species across different timescales. The specific calculations were performed using the following formulas:

$$\text{mRAI}=\frac{T_{i}}{N}\times 1000;\text{sRAI}=\frac{S_{j}}{N}\times 1000$$

Where *T*_*i*_ represents the number of independent detections of the Gongshan muntjac during the *i*‐th month (*i* = 1–12); *S*_*j*_ denotes the number of independent detections of the Gongshan muntjac in the *j*‐th season (*j* represents spring, summer, autumn, and winter); and *N* signifies the total number of camera days.

#### 2.3.5 Activity Patterns

To compare the daily activity rhythms and seasonal variations of the Gongshan muntjac, the seasons were defined according to the local climatic characteristics: spring is from March to May, summer is from June to August, autumn is from September to November, and winter is from December to February of the following year.

The kernel density estimation (KDE) method was employed to analyze the activity patterns of the Gongshan muntjac (Ridout and Linkie 2009; Meredith and Ridout 2014). This method assumes that the behavioral activities of the target species are continuously distributed in a 24 h cyclical pattern, with independent detections considered random samples drawn from this distribution (Widodo et al. 2022). The overlap coefficient (*Δ*) quantifies the similarity in activity patterns by measuring the degree of overlap between two kernel density curves, with values ranging from 0 (no overlap) to 1 (complete overlap). *Δ*1 was used for small sample sizes of less than 50 independent events, whereas *Δ*4 was used when both sample sizes were greater than 75. A value of *Δ* > 0.75 indicates a high degree of overlap, 0.50 < *Δ* ≤ 0.75 indicates moderate overlap, and *Δ* ≤ 0.50 indicates low overlap (Ridout and Linkie 2009; Monterroso et al. 2014). The overlap coefficient was estimated using smooth bootstrapping with 1000 iterations.

The analysis and plotting were conducted using the “overlap” and “activity” packages in R 4.3.3 (Meredith and Ridout 2014; Rowcliffe 2016). In the resulting plots, the *x*‐axis represents time, and the *y*‐axis (density) represents the probability of detecting the target species at that time point (Chen et al. 2019). The *p* value represents the variability of the activity rhythm in different samples, with *p* < 0.01 indicating that the activity data between the two samples were significantly different (Donini et al. 2025).

## 3 Results

From April 2023 to December 2024, a total of 52 deployed cameras recorded valid images and videos of the Gongshan muntjac, totaling 14,098 camera days. These recordings yielded 4846 images and videos, corresponding to 914 independent detections of the species.

### 3.1 Habitat Distribution

**FIGURE 3:**
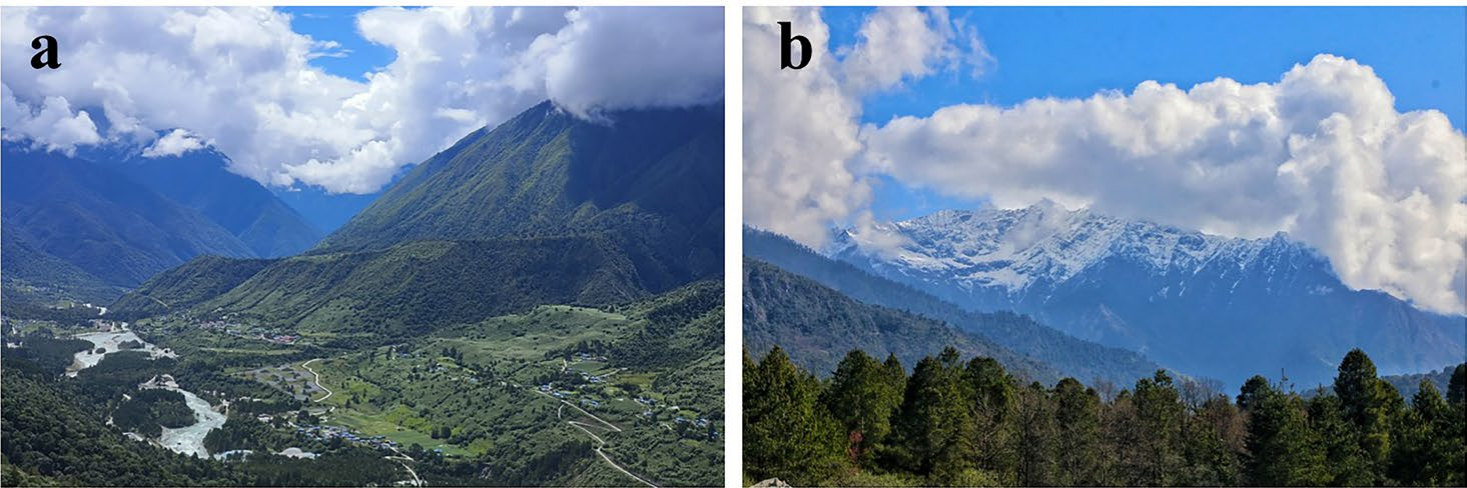
Photographs of (a) typical habitats and (b) unsuitable high alpine areas of *Muntiacus gongshanensis* in Gedang, Southeastern Tibet.

The Gongshan muntjac showed distinct patterns in its distribution across the two forest types. The primary habitats for the species were broadleaf and coniferous forests within the study area, with the highest activity observed in broadleaf forests (Table 1; Figure 3a). However, no activity by the species was recorded in shrubland or alpine vegetation in the high‐elevation areas of the region (Figure 3b).

**TABLE 1:** The capture rates of *Muntiacus gongshanensis* in different vegetation types.

Forest types	No. of camera sites	Camera days (d)	Images and videos	Independent detections	Percentage (%)
Broadleaf forest	47	12,686	4581	852	93.22
Coniferous forest	5	1412	265	62	6.78
Total	52	14,098	4846	914	100

### 3.2 Elevation Distribution

The Gongshan muntjac predominantly inhabited areas below 2800 m across various elevation ranges (99.13%), with particularly frequent habitat use below 2200 m (55.48%). Specifically, the highest concentration of activity was observed below 2000 m, which constituted 32.39% of the total number of independent detections. The second highest concentration was found between 2000 and 2200 m, accounting for 23.09% of the total number of independent detections. Furthermore, considerable habitat use was also detected in the elevation ranges of 2200–2400 m, 2400–2600 m, and 2600–2800 m, representing 11.38%, 17.94%, and 14.33% of the total independent detections, respectively. Conversely, the activity of Gongshan muntjac was markedly restricted in areas above 2800 m, with only 0.88% of the total number of independent detections occurring in this range (Table 2).

**TABLE 2:** Capture rates of *Muntiacus gongshanensis* at various elevations.

Elevation (m)	Number of camera sites	Camera days (d)	Images and videos	Independent detections	Percentage (%)
< 2000	9	2190	1988	296	32.39
2000—2200	13	3812	1186	211	23.09
2200—2400	6	1658	403	104	11.38
2400—2600	10	2595	654	164	17.94
2600—2800	11	2796	576	131	14.33
2800—3000	2	760	32	7	0.77
> 3000	1	287	7	1	0.11
Total	52	14,098	4846	914	100

### 3.3 Sex Ratio and Relative Abundance

Among the 914 independent detections of the Gongshan muntjac collected, 49 were excluded because of the inability to determine the sex of the individuals. Among the 865 sex‐identifiable independent events, 89 were group‐independent events, and 776 were individual‐independent events. The numbers of independent detections for females and males were 436 and 492, respectively, yielding a male‐to‐female ratio of 1.13:1. The male‐to‐female ratio fluctuated across seasons, with the highest ratio in winter at 1.43:1, followed by autumn at 1.12:1. In spring and summer, the ratios were slightly lower, at 1.06:1 and 1.09:1, respectively (Table 3).

**TABLE 3:** The number and sex ratio of male and female *Muntiacus gongshanensis* in each season.

Season	Independent detections (*S*_*j*_)	Number of female records	Number of male records	Male‐to‐female ratio	sRAI
Spring	205	103	109	1.06:1	14.54
Summer	307	142	155	1.09:1	21.78
Autumn	285	144	161	1.12:1	20.22
Winter	117	47	67	1.43:1	8.30
Total	914	436	492	1.13:1	64.83

**FIGURE 4:**
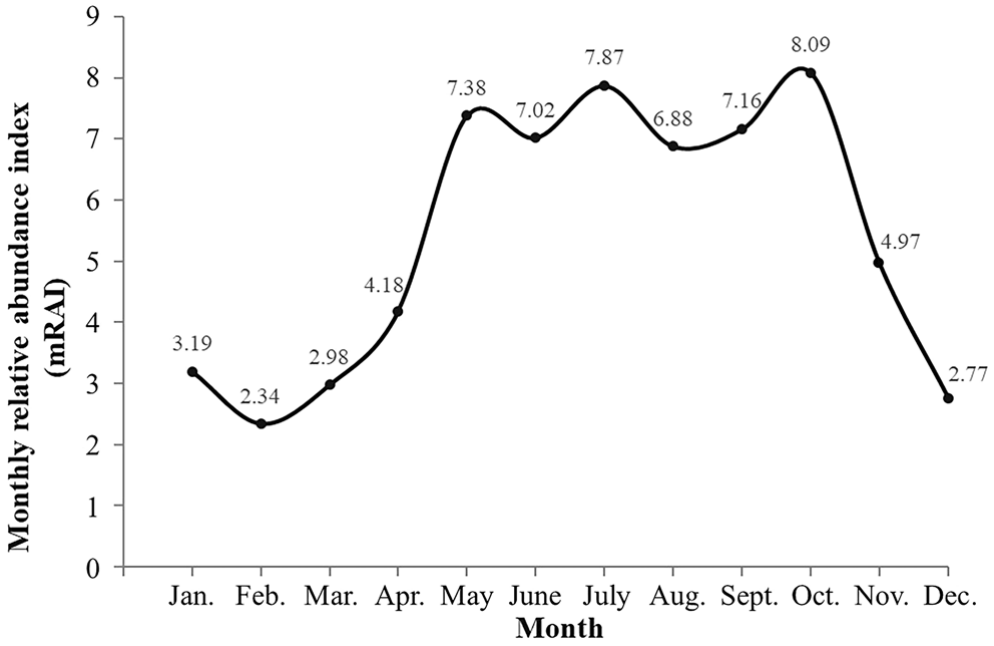
Monthly relative abundance index (mRAI) of *Muntuntia gongshanensis*.

In terms of the sRAI and mRAI of Gongshan muntjac, the relative abundance was highest in summer at 21.78, followed by autumn at 20.22, with a peak mRAI value of 8.09 in October. In contrast, the relative abundance was lowest in winter at 8.30, and the lowest mRAI value was 2.34 in February (Table 3; Figure 4).

### 3.4 Social Structure and Frequency

The social structure patterns of Gongshan muntjac were analyzed based on 865 sex‐identifiable independent detections, which were categorized into eight groups: solitary adult female (*n* = 349), solitary adult male (*n* = 427), female–male pair (*n* = 60), doe‐fawn group (*n* = 19), female group (*n* = 5), male group (*n* = 2), one male‐two female group (*n* = 2), and family group (*n* = 1) (Table 4). The proportions of these group patterns revealed that solitary individuals were the most prevalent, accounting for 89.71% of all independent detections. The female–male pairs were the second most frequent group, accounting for 6.94% of the detections. Other group patterns were relatively rare, collectively representing 3.35% of all detections: the doe‐fawn group represented 2.20%, the female group accounted for 0.58%, the family group made up only 0.12%, and both the male group and one male‐two female group each constituted 0.23%. Notably, in all the observed groups, the largest group size was three individuals, each containing only a single offspring, with no instances of multiple offspring being captured.

**TABLE 4:** Group types and percentages of *Muntiacus gongshanensis* recorded during the camera‐trapping survey.

Group type		Independent detections	Percentage (%)
Solitary individual	Solitary adult female	349	40.35
	Solitary adult male	427	49.36
Group of 2 individuals	Female–male pair	60	6.94
	Doe‐fawn group	19	2.20
	Female group	5	0.58
	Male group	2	0.23
Group of 3 individuals	One male‐two female group	2	0.23
	Family group	1	0.11
Total		865	100

### 3.5 Activity Patterns

**FIGURE 5:**
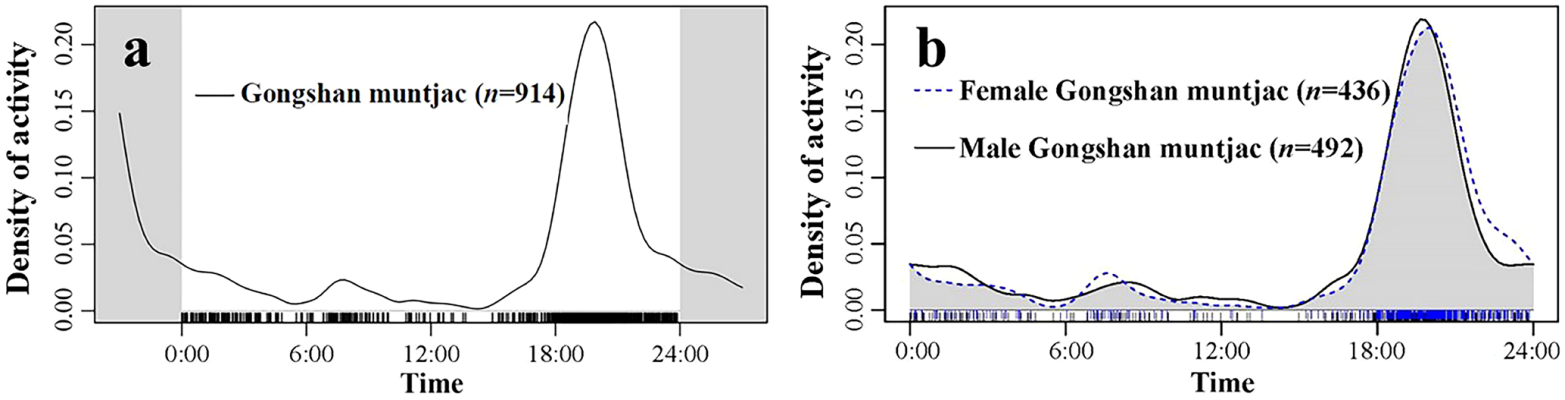
Daily activity rhythms of *Muntiacus gongshanensis* throughout the year (a) and overlap between solitary males and females (b).

The results demonstrated that the daily activity rhythms of the Gongshan muntjac exhibited a distinct bimodal pattern, with the main activity peak concentrated between 19:00 and 21:00 in the evening and a smaller peak between 07:00 and 09:00 in the morning. The initial recorded activity in the early morning commenced at 05:00, with a gradual increase leading to a peak at 08:00. The second peak began at 17:00, followed by a sharp increase in activity frequency after 18:00, culminating in a peak at 20:00. Conversely, a notable decrease in activity was observed from 04:00 to 06:00 and from 12:00 to 16:00, with two valleys of only three independent detections recorded at approximately 05:00 and 15:00, indicating the lowest activity levels (Figure 5a). The daily activity rhythms largely overlapped between female and male Gongshan muntjac, and the difference in their daily activity pattern curves was not significant (*Δ*4 = 0.917, *p* = 0.20) (Figure 5b).

**FIGURE 6:**
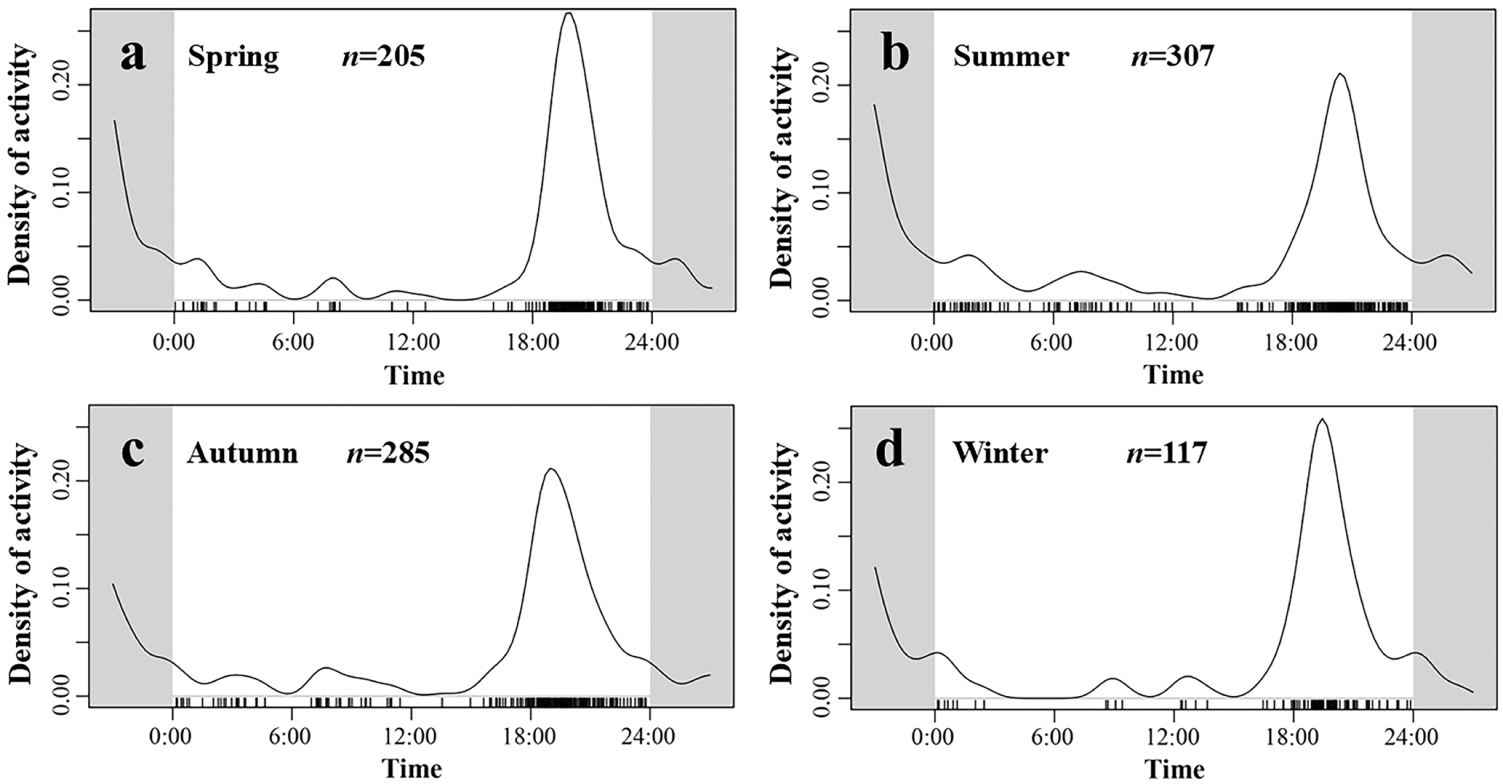
Seasonal variations in the daily activity rhythms of *Muntiacus gongshanensis* . (a) Spring; (b) Summer; (c) Autumn; and (d) Winter.

**FIGURE 7:**
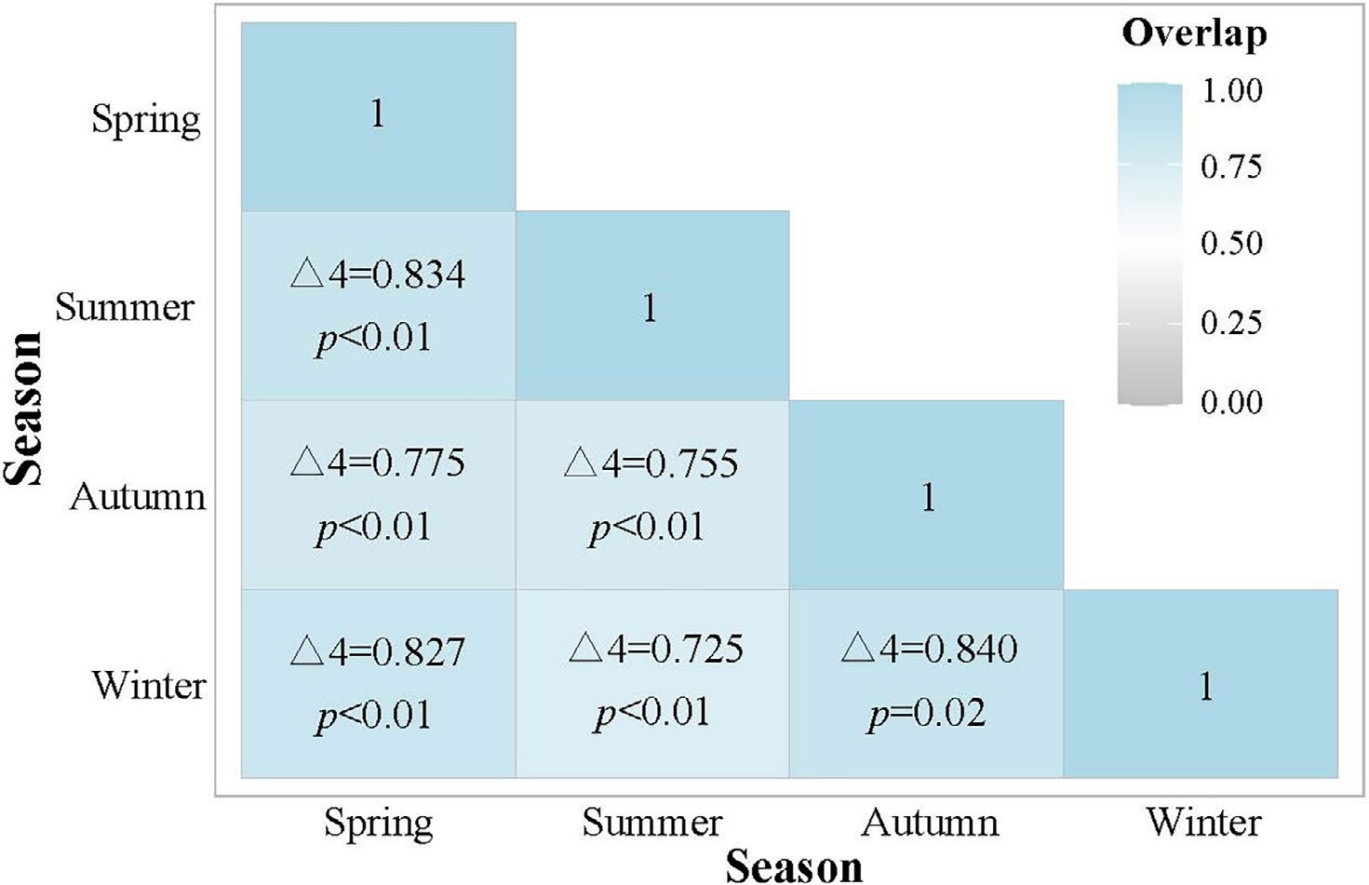
Coefficients of daily activity overlapping of *Muntiacus gongshanensis* across different seasons.

With respect to seasonal activity patterns, despite fluctuations in daily activity rhythms across different seasons, there was no significant variation in the intensity of seasonal activity. The peak activity times for both spring and winter were recorded at approximately 20:00 (Figure 6a,d). However, during summer and autumn, the peak activity times differed slightly, with the autumn peak moving earlier to 19:00 and the summer peak occurring later at 21:00 (Figure 6b,c). Furthermore, during winter, a slight active period was detected at approximately 13:00, whereas very low activity was observed from 03:00 to 07:00 (Figure 6d). The results of the overlap analysis of daily activity rhythms across different seasons for Gongshan muntjac were as follows: spring–summer (*Δ*4 = 0.835, *p* < 0.01); spring‐autumn (*Δ*4 = 0.776, *p* < 0.01); spring–winter (*Δ*4 = 0.828, *p* < 0.01); summer‐autumn (*Δ*4 = 0.754, *p* < 0.01); summer‐winter (*Δ*4 = 0.725, *p* < 0.01); and autumn‐winter (*Δ*4 = 0.840, *p* = 0.02). These findings indicated that the daily activity rhythms of Gongshan muntjac presented high overlap coefficients (*Δ*4 > 0.75) across different seasons except for summer and winter, which presented moderate overlap (0.50 < *Δ*4 ≤ 0.75), with the greatest overlap occurring between autumn and winter, and the differences between seasons were statistically significant (Figure 7).

## 4 Discussion

### 4.1 Muntjac Distribution and Social Patterns

The distribution of the Gongshan muntjac across various elevation ranges reveals critical insights for its conservation. Our study demonstrated that this species had the highest concentration of activity below 2800 m, primarily below 2200 m. Similarly, Deng et al. (2023) studied Reeves' muntjac in Jiande City and revealed that within an elevation range of 29–907 m, the species was predominantly concentrated between 200 and 399 m, with a significantly high detection rate that was approximately twice as high as that in other areas. Additionally, the detection rate of Reeves' muntjac initially increased but then decreased with increasing elevation, and Reeves' muntjac exhibited the highest habitat use in evergreen broadleaf forests. Concurrently, Reeves' muntjac exhibits seasonal migratory behavior (Zhang et al. 2012). In summer, they prefer to inhabit high‐elevation areas, but as the autumn and winter seasons advance, they progressively descend to lower elevation due to a reduction in and deterioration of food resources. In our study, although infrared cameras were strategically placed across a wide range of elevations, the activity of the Gongshan muntjac was extremely limited in the high‐elevation areas. The habitat use of the Gongshan muntjac is primarily in mid‐low elevation areas and predominantly in broadleaf forests, which appears to be a key characteristic of its distribution; thus, conservation efforts should focus on protecting and restoring these areas to ensure the long‐term sustainability of the species. This habitat preference likely indicates that broadleaf forests provide essential resources and protection for the species, including abundant food sources and shelter from predators, whereas harsh environments and sparse vegetation with limited plant diversity at relatively high elevations are less suitable for the survival and reproduction of the species (Bhatt et al. 2022; Salmanpour et al. 2025). Moreover, infrared camera‐trap footage reveals that the Gongshan muntjac is a highly agile and cautious species that often pauses to forage and frequently scans its surroundings. This behavior likely represents an adaptation to its dense forest habitat, where stealth and alertness are crucial for avoiding predators.

**FIGURE 8:**
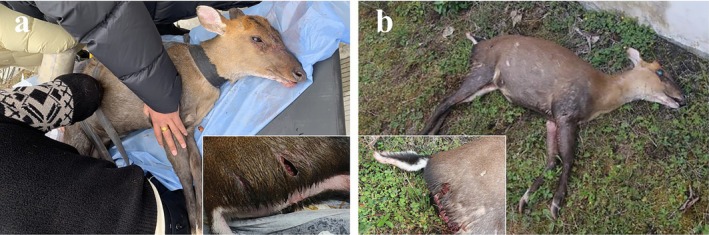
(a) A rescued female *Muntiacus gongshanensis* and (b) a deceased female *Muntiacus gongshanensis* discovered during patrol operations. Both were attacked by predators in their caudal pelvic regions.

In the present investigation, the number of Gongshan muntjac males was slightly higher than the number of females, with a male‐to‐female ratio of 1.13:1. Furthermore, the species predominantly engaged in solitary activities, constituting 89.71% of all independent detections. This pattern may be attributed to a complex interplay of environmental factors, resource partitioning, and genetic determinants. Moreover, the high male‐to‐female ratio likely intensifies competition among males, driving them to establish and defend territories to secure access to resources and mates (Thomas 2019), which may also partly explain the rarity of the male groups observed in our study. Notably, our prior study indicated that within the study area, the Gongshan muntjac frequently serves as a target for predation and displacement by other carnivores, such as jackals, and most rescued individuals were relatively small female Gongshan muntjac (Wang et al. 2024), leading us to hypothesize that the high predation rate of the more vulnerable female Gongshan muntjac may have contributed to the observed lower number of females in this study (Figure 8). Previous studies have demonstrated that habitat structure and predation pressures significantly influence the population dynamics and sex ratios of ungulates (Berger and Gompper 1999). Compared with males, ungulate females are often more vulnerable to predation, especially when provisioning offspring, during which they may select habitats that offer great protection from predators, even if these areas provide few food resources; otherwise, they may opt for areas with rich resources despite the increased predation risk (Kojola et al. 2021; Blum et al. 2023). From another perspective, infrared cameras, unlike direct observations, may not fully capture entire animal aggregations because of their triggering mechanism, limited monitoring angles, range constraints, and environmental factors, such as vegetation, and terrain. For example, if an animal aggregation extends beyond the camera's range or is partially obscured, the camera may capture only a portion of the group. This limitation may result in an overestimation of the proportion of solitary individuals when the sex ratio and group patterns are analyzed on the basis of infrared camera data. However, given the exceedingly high proportion of solitary groups among the Gongshan muntjac, the impact of this bias is considered minimal in our study. In support of our findings, Chen et al. (2022) reported a similar sex ratio of 1:1.13 (male‐to‐female ratio) and a predominance of solitary individuals living in Reeves' muntjac in the Minshan area, with solitary groups constituting 91.97% of the total detections. These findings suggest that, like Reeves' muntjac, Gongshan muntjac predominantly leads a solitary lifestyle, which is closely linked to local food availability and intraspecific competition, as well as the complex influences of other environmental or ecological factors, such as habitat structure or predator density.

### 4.2 Muntjac Activity Patterns

The relative abundance of Gongshan muntjac, as measured by the sRAI and mRAI, exhibited significant seasonal variation. The highest abundance was observed in summer and autumn, with a peak mRAI in October. Conversely, the lowest abundance occurred in winter, and the lowest mRAI was recorded in February. The daily activity rhythms of wildlife reflect their adaptive patterns in response to factors, such as habitat, climate, and coexistence with sympatric species within their activity range. Extensive research has demonstrated that the daily activity rhythms of muntjac species typically exhibit a bimodal activity pattern, with distinct crepuscular habits. For example, the daily activity peaks of Red muntjac are concentrated mainly from 06:00 to 10:00 and from 16:00 to 20:00, with peak times varying seasonally (Wang et al. 2015). The activity peaks of Black muntjac occur from 07:00 to 09:00 and from 16:00 to 18:00 (Zhang et al. 2012; Chen et al. 2021). Reeves' muntjac is most active during the periods from 06:00 to 09:00 around sunrise and from 17:00 to 19:00 around sunset, with its activity patterns jointly influenced by natural factors, such as altitude, temperature, and slope, as well as anthropogenic disturbances (Ma et al. 2024; Zhang et al. 2024). These rhythms are likely closely associated with food acquisition, predator avoidance, and social interactions (Chen et al. 2022; Rodríguez‐Luna et al. 2024). The daily activity rhythms of the Gongshan muntjac mirror those of the aforementioned species, with primary activity peaks concentrated in the early morning from 07:00 to 09:00 and in the evening from 19:00 to 21:00, suggesting that these species occupy closely related ecological and evolutionary niches.

Research has confirmed that, with the emergence of tender leaves and shoots preferred by Reeves' muntjac in spring, their activity intensity correspondingly increases. Food availability is a key factor influencing the annual activity patterns of Reeves' muntjac (Zhang et al. 2024). However, although the activity intensity of the Gongshan muntjac varies seasonally, the overall variation trend of its daily activity rhythms is not significant, with high overlap coefficients observed across all seasons except between summer and winter. This phenomenon may be closely related to the climatic characteristics of the study area. Unlike the plains or hilly regions where Reeves' muntjac typically live, our study area is situated in the subtropical humid climate zone on the eastern slopes of the Himalayas, where vegetation is abundant and the annual temperature variation is not large, leading to relatively stable temperatures and food availability year‐round. Consequently, the Gongshan muntjac does not exhibit pronounced seasonal migratory behavior or significant changes in its activity patterns. These results highlight the distinct activity patterns and temporal variations of the Gongshan muntjac, which are essential for guiding conservation efforts.

### 4.3 Muntjac Taxonomy and Conservation

The species in the genus *Muntiacus* are difficult to distinguish because of their similar antler shapes and body sizes, among other basic morphological characteristics, which often lead to taxonomic confusion and pose a challenge to their biodiversity conservation. This ambiguity in species definition and diagnosis often leads to the misclassification of common species as endangered, whereas genuinely threatened species may be inaccurately categorized as least concern or, in some cases, entirely overlooked (Hong 2016). Ongoing updates to the distribution ranges of muntjac species further complicate classification, with the status of certain groups as independent species still under debate (James et al. 2008; Le et al. 2014). A prime example of this dilemma is the Gongshan muntjac, which was previously misclassified as either Fea's muntjac or a western subspecies of the Black muntjac (Smith and Xie 2009; Liu et al. 2022). However, despite its later accurate classification as a species, the Gongshan muntjac has been recognized as one of the least understood ungulate species thus far (Ma et al. 1990; Liu et al. 2022).

In the IUCN (2025) Red List, the majority of genus *Muntiacus* are classified as either Least Concern (LC) or Data Deficient (DD). Specifically, the Southern red muntjac ( *M. muntjak* ), Northern red muntjac, and Reeves' muntjac are categorized as LC, whereas the Gongshan muntjac, Fea's muntjac, Leaf muntjac, Roosevelts' muntjac ( *M. rooseveltorum* ) and Annamite muntjac ( *M. truongsonensis* ) are categorized as DD. This classification has resulted in a chronic lack of academic attention to such species (Wurster‐Hill and Seidel 1985; Le et al. 2014; Li et al. 2017; Zhang et al. 2021). However, evidence suggests that the actual number of Gongshan muntjac individuals has shown a declining trend, and China's Vertebrates Red List has upgraded the status of this species to Critically Endangered (CR) (Timmins and Duckworth 2016; Jiang et al. 2016). This reclassification underscores the urgent need for enhanced ecological and taxonomic research to inform the establishment of appropriate conservation strategies. In this study area, the relatively high abundance of Gongshan muntjac underscores the importance of this effort. Future research should focus on long‐term monitoring to better understand population dynamics and the impacts of conservation measures. Additionally, further taxonomic studies are needed to clarify the status and ensure accurate conservation prioritization.

## 5 Conclusion

This study comprehensively investigated the distribution, habitat preferences, social structure, and activity patterns of the Gongshan muntjac ( *Muntiacus gongshanensis* ) in the mountainous regions of Southeastern Tibet. The results underscore the importance of broadleaf forests below 2800 m as vital habitats for the species, necessitating prioritization in conservation efforts for protection and restoration. By comparing these ecological traits with those of other muntjac species, similarities and differences were identified to enhance the understanding of the population distribution and activity patterns of the Gongshan muntjac. Moreover, regular long‐term camera trap monitoring should be implemented to effectively assess species status, evaluate conservation strategies, and guide resource allocation, thereby ensuring adaptability to environmental changes and population dynamics. These findings not only shed light on the ecological characteristics of this species, which has yet to be assessed by the IUCN due to scarce information, but also establish a scientific basis for assessing habitat preferences and understanding the practical applications of environmental influences on the Gongshan muntjac. Furthermore, the results underscore the importance of our research in providing actionable recommendations, offering crucial insights into its conservation and management, and serving as a template for studying other rare and elusive ungulates.

## Author Contributions

**Qianqian Wang:** conceptualization (lead), data curation (lead), formal analysis (lead), investigation (equal), methodology (lead), project administration (supporting), visualization (lead), writing – original draft (lead), writing – review and editing (lead). **Biao Yang:** conceptualization (equal), funding acquisition (lead), methodology (supporting), project administration (lead), resources (equal), writing – original draft (supporting), writing – review and editing (supporting). **Jin Chang:** formal analysis (supporting), visualization (supporting), writing – original draft (supporting). **Xin Wang:** investigation (equal), project administration (supporting). **Xiaoguo Chen:** data curation (supporting), formal analysis (supporting), investigation (supporting). **Shilin Li:** investigation (supporting). **Jiangcuo Renzeng:** investigation (supporting). **Dunzhu Gongqiu:** investigation (supporting). **Li Zhang:** resources (supporting), writing – review and editing (supporting).

## Ethics Statement

This study was permitted with approval from both the Forest and Grassland Bureau of Linzhi Municipal and the Forest and Grassland Bureau of Medog County.

## Conflicts of Interest

The authors declare no conflicts of interest.

## Supporting information


Data S1

## Data Availability

All the data and materials generated or analyzed during this study are included in this published article and its Supporting Information files.
